# The importance of multiparasitism: examining the consequences of co-infections for human and animal health

**DOI:** 10.1186/s13071-015-1167-9

**Published:** 2015-10-20

**Authors:** Elise Vaumourin, Gwenaël Vourc’h, Patrick Gasqui, Muriel Vayssier-Taussat

**Affiliations:** UR346 Animal Epidemiology Research Unit, INRA, Saint Genès Champanelle, France; USC BIPAR, INRA-ANSES-ENVA, Maisons-Alfort, France

**Keywords:** Multiparasitism, Interactions, Associations, Mechanisms, Observations, Modelling

## Abstract

Most parasites co-occur with other parasites, although the importance of such multiparasitism has only recently been recognised. Co-infections may result when hosts are independently infected by different parasites at the same time or when interactions among parasite species facilitate co-occurrence. Such interactions can have important repercussions on human or animal health because they can alter host susceptibility, infection duration, transmission risks, and clinical symptoms. These interactions may be synergistic or antagonistic and thus produce diverse effects in infected humans and animals. Interactions among parasites strongly influence parasite dynamics and therefore play a major role in structuring parasite populations (both within and among hosts) as well as host populations. However, several methodological challenges remain when it comes to detecting parasite interactions. The goal of this review is to summarise current knowledge on the causes and consequences of multiparasitism and to discuss the different methods and tools that researchers have developed to study the factors that lead to multiparasitism. It also identifies new research directions to pursue.

## Background

More than 80% of all known species, from a multitude of taxa, are considered to be parasites, that is, organisms that depend on a host to survive [1]. Parasites, broadly defined, do not necessarily provoke pathogenic effects in their hosts, their degree of pathogenicity depends on host-related environmental conditions [2]. However, for many parasites, the pathological impact on their host species, especially wild species, is unknown and has not been investigated (see for example [3, 4]). Furthermore, the reality, more or less ignored until recently, is that most parasites co-occur with other parasites [5–7]. Parasites regulate the populations of a large number of host species found across diverse ecosystems and make a significant contribution to biodiversity [8, 9]. In humans alone, more than 1,400 pathogen species have been described, including viruses, bacteria, helminths, protozoa, and fungi [10]. As many as 30% of infections may be co-infections, and this rate can climb as high as 80% in certain human populations [11].

Consequently, organisms can be viewed as ecosystems—communities of living creatures associated with particular environmental conditions—and may thus form a type of co-evolutionary network [12–14]. Co-infections may result when hosts are independently infected by different parasites at the same time or during a sequential infection as well as when interactions among parasite species facilitate co-occurrence (*e.g.* when one parasite induces an immune defect allowing subsequent infections by other parasites). Such interactions can have important repercussions on human or animal health because they can alter host susceptibility to other parasites, infection duration, transmission risks, clinical symptoms and consequently treatment and prevention strategies. Interactions may be synergistic, *i.e.* the presence of one parasite may facilitate subsequent infections by other parasites; or antagonistic, *i.e.* the presence of one parasite may inhibit subsequent infections by other parasites. Co-infections may also result from common risk factors, which can generate a purely statistical association among parasites. In other words, certain parasites may co-occur more frequently than expected simply because the same factors promote their presence, not because they are interacting synergistically. Such factors may include environmental conditions, climatic conditions, host density levels, host behaviours, or host physiological conditions [11, 15–17].

It is interactions, rather than associations, among parasites that play a major role in structuring both parasite populations (both within and among hosts) and host populations [11, 18]. However, several methodological challenges remain when it comes to understanding these interactions, in particular, detecting interaction among associations [19, 20] and understanding interactions in the complexity of natural systems [21, 22]. Furthermore, parasites are often viewed as engaging in one-on-one interactions, which are most commonly modelled using pairwise interaction networks, such as those used in ecology [23, 24]. Historical and current microbiological research focuses mainly on a single genera or group of parasites (*e.g.* virus, bacteria, fungi) and on the descriptions of metabolic pathways or gene expressions rather than a broader approach to multi-parasitism. However, it seems more likely that parasites are interacting in groups (*e.g.* [25]). The problem is that such a situation is difficult to model, because the number of possible interactions grows with the number of parasites [11].

The goal of this review is to summarise current knowledge on the causes and consequences of multiparasitism and to discuss the different methods and tools that researchers have developed to study the factors that lead to multiparasitism. It also identifies new research directions to pursue.

## What drives multiparasitism?

### Host characteristics facilitate co-infections

As mentioned above, parasites may co-occur more frequently than expected by chance because of common risk factors as opposed to synergistic interactions. In particular, two sets of ecological factors may promote multiparasitism: 1) factors that influence host exposure, namely the spatial distribution of hosts and 2) factors that influence host susceptibility are intrinsic to hosts, namely host life-history traits.

#### Co-infections due to host distribution patterns and use of space

The spatial ecology of the environment in which hosts and parasites occur plays a crucial role in host exposure [26]. In tropical latitudes the combination of a higher diversity of free-living species with specific abiotic factors (*e.g.* rainfall, hygrometry, soil moisture) seem to favour higher parasite diversity in humans [27] and in some wild mammals [28] (see examples of other patterns in mammal species in [29]). Also, hosts with larger distributions are more likely to become co-infected, as are hosts that occupy ecological niches in which several parasites are present [30]. Consequently, generalist species, which can tolerate a wide range of environmental conditions and exploit a large number of resources, are exposed to a greater diversity of parasites (*i.e.* the area–species diversity relationship: [31]). Some rodent species, for example, are ubiquitous and may thus serve as bridges between many different environments and parasite populations. As a consequence, some rodent species have higher parasite loads and have been described as “good vessels” for parasites [32, 33]. At present, the world is growing smaller as a result of expanding transportation networks (planes, boats etc. allowing the transport of vectors such as mosquitoes) and the effects of globalisation (greater movement of humans and goods including the smuggling of animals and plants). As a consequence, hosts are facing greater and greater levels of exposure.

#### Co-infections due to host life-history traits

An individual’s life-history traits are those characteristics that enhance the production and survival of offspring [34–36] they are constantly being shaped by natural selection. Key life-history traits include growth rate, lifespan, fecundity, parental investment, and investment in immune defence. Environmental conditions have a major influence on life-history traits, primarily by imposing trade-offs, such as the trade-off between growth and reproduction. One way that environmental conditions affect life-history traits is through their impacts on host physiology and susceptibility to parasites. For instance, stressed or malnourished hosts are more likely to become infected [37].

In turn, host life-history traits could influence the occurrence of multiparasitism in numerous ways. Many studies have investigated the links between life-history traits and parasite richness at the level of host species on different major determinant [24, 38]. However, the study of the relationship between host life-history traits and parasite co-infections (or co-occurrence) is at its infancy. We can only infer a potential positive link between a high parasite species richness (or infection prevalence) and the probability to be co-infected more frequently. At the individual host level, life history traits, such as parental investment, have been shown to favour co-infection [39]. The relationship between lifespan and multiparasitism has been also investigated at specie level. At the host specie level, it has been shown that longer lived hosts are less multiparasited. This is because they tend to invest more in anti-parasite defence [40]. We could infer that individuals of long lived species are less co-infected. In addition, whether, within species, long lived individuals may be less co-infected remains to be investigated. The degree of host sociality may also play a major role on the probability to be co-infected. The hosts living in large groups could be more parasited (stronger transmissions and higher parasite species richness) and so more co-parasited. However, social species may have evolved specific behaviours to counter parasite, thus, social species could be less co-parasited [41].

Hosts may deal with infection by increasing their resistance (*i.e.* the ability to limit parasite burden) or their tolerance (*i.e.* the ability to limit the harm caused by a given burden) according to their investment in immunity [42, 43]. Tolerance may ease multiparasitism as it seems to be the case for eel [44]. This investment can be affected by many factors, in particular, by a trade-off between reproduction and immune defence, such as seen in introduced species [45] or in certain males. For example, in bank voles, testosterone acts as an immunosuppressant and affects acquired resistance to ticks and tick-borne pathogens (*i.e.* Lyme spirochetes [46, 47]).

The properties of host species to be natural competent reservoirs are linked to intrinsic host traits associated for example with life history features and host population density and vary between the different pathogens [48].

### Parasite characteristics facilitate co-infections

The characteristics of parasites that facilitate multiparasitism may be general in nature or specifically tied to different mechanisms that underlie host-parasite interactions.

#### General characteristics

Like their hosts, different parasite species will come in contact with a narrower or wider range of other parasites depending on how broadly they are distributed. The common roundworm (*Ascaris lumbricoides*) provides a perfect example. Because this species infects more than 25% of the human population [49], it is not surprising to discover that it co-occurs with 47 other species of helminths or protozoa [50]. Different parasites are also more likely to come into contact with each other when they have overlapping ecological niches.

#### Parasite characteristics related to infection mechanisms

##### Infection mechanisms related to gaining entry into the host

Parasites gain entry into hosts primarily using mechanical mechanisms [51]: they essentially open an entryway for themselves, which can also be used by other species. For example, certain endoparasites cause lesions in mucous membranes, making it easier for other parasites to infect the host; for instance, in humans, infection by the herpes simplex virus type 2 (HSV-2) paves the way for HIV infection [52–58].

##### Infection mechanisms related to host exploitation

Infection mechanisms related to host exploitation by parasites are essentially competition mechanisms, directly or indirectly [11, 59–61].

Competition may occur between two parasite species because they occupy the same physical space within their host [62, 63]. If the parasites modify their respective ecological niches as a consequence, they may ultimately be able to coexist [64]. In the case of helminths, for example, attachment sites vary depending on which other parasites are present [59, 65, 66].

Competitive interactions may also affect species abundances [67–70], as well as parasite loads [21], transmission [71–73], and virulence [74]. Competition may occur at the molecular level; for example, certain parasites produce toxins (*e.g.* bacteriocins, nitric oxide) that can reduce or enhance the growth and virulence of fellow parasites [75–79]. Bacteria in the genus *Enterobacter*, which are part of the intestinal microbiota of *Anopheles* mosquitoes, make their hosts resistant to infection by *Plasmodium falciparum* [80], a malaria parasite of major importance to human health. Competition between two parasites can result in one parasite “sabotaging” the other’s efforts to control host behaviour, which can increase host vulnerability to predators; this scenario has been observed in a system involving amphipods that are co-infected with a nematode and a trematode [81, 82].

Competition can also have an inhibitory effect on growth [83–85], a phenomenon that has been named “the Jameson effect” [86]. In this situation, competing parasites are negatively affected by a decline in available resources [87]. For example, when hosts become anemic as a result of infection with helminths, the worms begin competing with microparasites that require red blood cells [88]. When parasites co-occur within a single host, gene exchange may take place (*e.g.* via plasmid transfer or recombination) and hence may be a powerful driver of pathogen evolution. Such exchanges may result in functional changes that make parasites more virulent or resistant (*e.g.* resistance to antibiotics, transfer of pathogenicity islands [89–91]).

##### Infection mechanisms related to host response

Each of the parasites present within a host must be able to confront the host’s immune defences, particularly its immune memory. Specifically, parasites interact with the molecules produced by the host’s immune system, such as antibodies, cytokines, and complement system proteins [26, 92]. Parasites can suppress a host’s immune system, for example, by driving down levels of lymphocytes and certain interleukins. Such effects can make it easier for other parasites to infect the host [61]. In some cases, parasites act beyond. For example, it has been highlighted in children that the measles virus could suppress immune defences against nonmeasles infectious diseases which have been encountered previously (*i.e. “immune amnesia”* [93]). However, when parasites interfere with a host’s immune system, there may also be an increase in the production of certain molecules, such as interleukins and antibodies that ultimately enhances immunity. Furthermore, immunity acquired against one type of parasite may be effective against other, antigenically similar parasites. The term cross-immunity is used to describe this phenomenon (*i.e.* when two similar antigens are targeted by the same antibodies and T cells). The benefits of cross-immunity have been observed in rabbits infected with different intestinal helminth species: *Trichostrongylus retortaeformis* negatively affects *Graphidium strigosum* via the host’s immune system [21]. The influence of the phenomenon of autoimmunity (*i.e.* immune responses of an organism against its own cells and tissues) on multi-infections may also be mentioned. Indeed, by disrupting the host immune response to infections, it can have an impact on the frequency of co-infections [94, 95].

Another mechanism related to the host’s immune system involves a polarisation of the immune response. In this case, there is a trade-off between two of the host’s cellular responses (*i.e.* between the production of Th1 and Th2 cells), which leads to different dynamics depending on whether the infection is caused by a single parasite or multiple parasites. This mechanism could therefore lead to dynamic interactions among parasites [61]. The polarisation of the immune response may benefit certain parasites while negatively affecting the host. This phenomenon can be observed in hosts that are co-infected by *T. cruzi* and various helminth species. *T. cruzi* activates the Th1 pathway, while the helminths activate the Th2 pathway [96]; the result is a trade-off that ultimately tips in *T. cruzi*’s favour, thus enhancing its transmission [97–99]. However, there are also cases in which the host benefits and the parasites are negatively impacted. For example, in individuals suffering from malaria that are also infected with helminths, the immune system can operate at an intermediary position along the trade-off curve, at which both parasites are sufficiently under control. The immune response directed at the malaria parasite (the Th1 pathway) is thus weaker, reducing the risk of neurological complications due to excessive levels of Th1 cells [100, 101].

## Methods used to study multiparasitism

Multiparasitism is mainly studied using data obtained from natural systems or under controlled laboratory conditions. These data may be analysed using exploratory models or mechanistic models. Additionally, it is possible to investigate the properties of these models using simulations.

### The origin of multiparasitism data

#### Data obtained from natural systems

Longitudinal studies are one of the main methods used to study multiparasitism in natural systems. This approach has numerous constraints; the most significant is the necessity of following study subjects over time. Individuals can be marked with simple, unique tags, as they are in Capture-Mark-Recapture (CMR) studies (*e.g.* [102, 103]) or with more technologically advanced devices, such as Argos transmitters or GPS chips (*e.g.* [104]). However, such systems are not well suited to tracking small animals, such as arthropods, which can act as vectors and are therefore of major epidemiological importance. In longitudinal studies, sampling techniques must never affect host fitness; consequently, only non- or minimally invasive methods should be used (*e.g.* sampling of blood, faeces, skin), thus the overall spectrum of parasites cannot be studied. Thanks to the temporal information yielded by these studies, it is possible to test whether the presence of one parasite affects the infection probability [105], persistence [106], and transmission [107] of other parasites. Such studies require substantial resources, in terms of money, personnel, and time.

Cross-sectional studies examine groups of individuals over short periods of time and yield population-level data. The nature of cross-sectional studies means that their sampling techniques can be more or less invasive. It is nonetheless possible to adapt cross-sectional studies to obtain longitudinal data [7, 108, 109]. For instance, a subset of individuals within a study population may be sampled at different moments in order to follow the population over time. If this design is used, sampling must not affect host fitness. Cross-sectional studies are less time-intensive and less expensive than longitudinal studies. They can examine a large number of hosts and are particularly helpful in studying host-parasite systems that involve emerging diseases, for which data are often limited [110–112].

#### Data obtained from laboratory experiments

Data obtained from laboratory experiments (*i.e.* under controlled conditions) can be used to test hypotheses generated from data on interactions in natural systems; they can also be used to tease apart the mechanisms underlying these interactions (*e.g.* cross-immunity, macrophage production, resistance, tropism), which can be difficult to do in natural systems (*e.g.* [113, 114]). Laboratory experiments therefore have an important role to play in studying the effects of multiparasitism and highlighting the synergistic and antagonistic interactions that take place among the diverse parasite groups (*e.g.* helminths, protozoa, fungi, viruses, acarians) that infect different host taxa, including mammals [115–117], insects [118, 119], and plants [120]. The advantage of experimental studies is that it is possible to control both the factors of interest (*e.g.* common risk factors), and to study a variety of relevant parameters (*e.g.* host growth, parasite establishment). However, findings obtained in the lab may be difficult to interpret because the results produced by experimental infections may differ from those produced by natural infections [121]. Another limitation is the fact that experiments are run in biological models (*e.g.* mouse, rat) that may not be receptive to parasite of non-model species (*e.g.* human, domestic and wildlife species).

### Analytical approaches

Two major approaches can be used to analyse multiparasitism data obtained from field and laboratory studies: exploratory models and mechanistic models.

#### Exploratory models

Exploratory models, whether empirical or descriptive in nature, mainly serve to analyse parasite co-occurrence; they cannot yield firm conclusions regarding parasite interactions. The parameters upon which they are based generally do not explicitly account for the biology of the study organisms. Exploratory models have the following two main advantages: they are fast and simple to use and they can be used on data from cross-sectional studies.

Multivariate analyses are one of the main exploratory approaches used to examine multiparasitism; common analyses include factor analysis/principal components (FA/PCA), discriminant analysis (DA), correspondence analysis (CA) and principal coordinates analysis (PCoA) [122]. These types of models assess whether parasites have a tendency to form clusters (*i.e.* they identify patterns of overdispersion). The disadvantage is that they generally do not have associated statistical tests (although see permutation tests, for example [123, 124]), which means that there is no statistical means of determining whether parasite co-occurrence deviates from what would be expected by chance.

The chi-square test is the most popular and straightforward of the different statistical tests that may be used to deal with multiparasitism data. It is mostly utilised to test for patterns involving two parasite species, but modified versions of the test have been developed that can deal with a greater number of species [125–127] or account for common risk factors that could influence parasite co-occurrence (*e.g.* [128]). The main disadvantage of this statistical approach is that it requires at least five individuals in each infection category. General linear models (GLMs) are also commonly employed. More specifically, multinomial logistic regression is best suited to multiparasitism data [129–131]. GLMs can explicitly take into account potential risk factors that are identified beforehand.

Over the last few years, methods derived from network theory have become more popular in ecology [132] and also offer an interesting approach for depicting relationships among multiple parasites [133–135]. They calculate association indices such as connectance [136], nestedness [137], and betweenness [138]. One drawback is that networks composed of fewer than 10 parasite species yield results that are difficult to interpret [139]. However, when more than 10 parasite species are included, the results will make biological sense. One difficulty is that, at present, statistical tests for association indices are poorly developed.

Association screening is a method that involves identifying parasite combinations [140]; compared to other methods, it has the advantage of being able to statistically determine whether parasites are associated. However, this approach can only include a limited number of parasites, depending on sample size and prevalence because the number of parasite combinations grows exponentially with the number of parasites.

#### Mechanistic models

Mechanistic models, such as deterministic models or probabilistic models, are used to study, in greater detail, the mechanisms underlying parasite associations and thus allow researchers to focus on potential interactions. They make it possible to study several issues related to multiparasitism, including the consequences of microparasite-macroparasite co-infections [141], the evolution of virulence [142], transmission dynamics [143], the role played by host life-history traits [144], and the effect of cross-immunity on co-occurring parasites [145]. They are generally more complex than exploratory models, and their structure is highly dependent on the specific issue under consideration. They are also more powerful and therefore yield more information about the processes being studied.

Deterministic models, such as SIR (Susceptible-Infected-Removed) models, use compartments to model biological systems. They are frequently used in epidemiological modelling and are built, for the most part, using data obtained in longitudinal studies [146]. They are more generally referred to as Multi-State-Models, or MSMs [147–149]. Because they explicitly incorporate time, these models can reveal changes in the flow of individuals among different compartments, regardless of whether a transient or steady-state process is being studied. It is therefore possible to test, for example, if the presence of one parasite affects the infection probability, persistence, or transmission of a second parasite [150]. Furthermore, deterministic models can be structured in different ways to test different hypotheses; for instance, they can be used to examine how co-infections impact host resistance or, conversely, how host age or number (*i.e.* host demography) impacts co-infections. However, these models may be complex because they require extensive parameterisation. Parameter estimates may be obtained from the literature, or models may be accompanied by simulations [151]. If a model’s parameters are at least partly characterised beforehand, Bayesian methods can be used to estimate parameter values [152, 153]. A few different types of deterministic models have been developed to explore questions related to multiparasitism. For example, Zhang *et al.* [144] built an SIR-type model to specifically test certain hypotheses regarding the influence of parasite interactions on the establishment and persistence of the different parasites present. Another type of model has been developed that takes into account the long-term immune memory that a parasite may induce. Gökaydin *et al.* [145] modelled the transmission dynamics of influenza A virus subtypes using an SIRI (Susceptible-Infected-Recovered-Infected) model, in which the rate of reinfection was reduced because infection with a different subtype conferred partial immunity. The authors emphasised that the reinfection threshold played an important role in regulating parasite diversity.

Like deterministic models, probabilistic models explicitly represent a system’s biology and can be used to estimate parameters and test different sets of hypotheses. However, in contrast to deterministic models, probabilistic models do not incorporate temporal dynamics; they are better suited to dealing with processes that have reached a steady state. These models are primarily focused on the states of individuals within a population and the likelihood that they will transition among states. The parameters they use can be estimated in the field (*e.g.* the infection probabilities associated with each parasite type found within a study population) [154]. There are as many types of probabilistic models as there are types of deterministic models, given that they are uniquely structured to specifically address hypotheses established *a priori.* Probabilistic models can use Markov-chain methods (*e.g.* [105, 107]) and diverse probability distributions (*e.g.* binomial, multinomial).

#### Simulations

Simulations use computer-generated data to study the properties (*i.e.* sensitivity or power) of exploratory and mechanistic models [155–157]. To do so, it is necessary to choose or generate a statistical test, as well as an associated null distribution (*i.e.* the distribution expected if only chance is in operation). Power analyses are useful when comparing different methods. Building a simulation that makes it possible to differentiate among alternative hypotheses is difficult in both theory and practice because, due to variability in the intensity of parasite interactions and associations, an infinite number of scenarios exist.

The specific research questions of interest will determine the sampling protocol and statistical analyses used. Recently, analytical tools developed in community ecology have been applied in other fields, such as invasion biology [158] and ecotoxicology [159]. These methods are facilitating the study of parasite communities. For example, parallels among predator-prey systems (described using Lotka-Voltera equations), host-parasite systems, and parasite-parasite systems have already been noted [160–163].

## The epidemiological and human and animal health consequences of multiparasitism

**Fig. 1:**
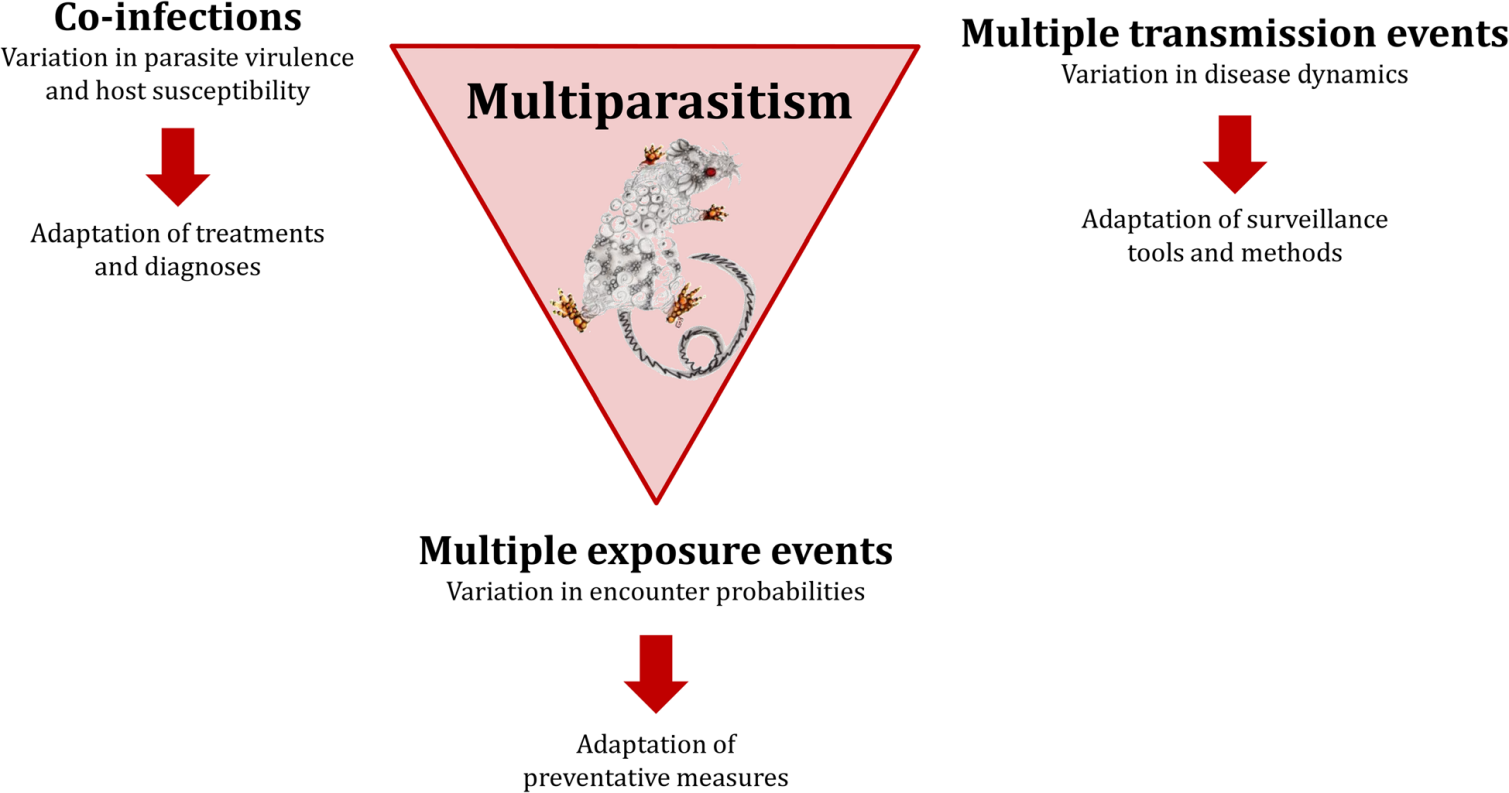
Possible effects of multiparasitism and the potential responses

The effects of multiparasitism originate in the interactions taking place among parasites within hosts. They are not simply the sum of the effects caused by each individual parasite; instead, they are the product of a combination of known and novel effects acting on key epidemiological parameters (*e.g.* [164–167]). An analysis that fails to take these interactions into account will yield a false representation of reality, especially in relation to individual parasites [61, 134]. This misrepresentation will have consequences from both an epidemiological standpoint, notably with regards to the incidence of infectious diseases, and from a medical standpoint, when it comes to the diagnosis, treatment, and prevention of infectious diseases. We will now address these different points using concrete examples that illustrate the following: 1) co-infections have an influence on the symptoms, duration, and treatment of infectious diseases; 2) it is crucial to take into account the manner in which co-infections influence parasite transmission to understand variation in infectious disease incidence and to obtain proper diagnoses; and 3) it is necessary to account for the consequences of host exposure to multiple parasites to develop effective disease prevention measures (see Fig. 1).

### Effects of co-infections on the symptoms (*i.e.* severity), duration, and treatment of infectious diseases

Co-infection can have negative effects on the host, ranging from the presentation of atypical symptoms to accelerated mortality. Studies in humans have linked helminth co-infections to enhanced morbidity for other infectious diseases, such as tuberculosis and HIV [168, 169]. Co-infection in addition to alter the likelihood of parasite establishment, growth and shedding of involved parasites, can generate super-shedders (*i.e.* individuals that for a period of time yield many more infective stages than most other infected individuals of the same host species, [170]) [171]. During co-infection, one parasite can be a driver of outbreaks of other parasites [172]. For example, it has been demonstrated that in zebra gastrointestinal helminths alter the dynamics of anthrax (*Bacillus anthracis*) by rendering hosts less capable of mounting effective anti-anthrax immune responses during the wet season [173].

Alternatively, co-infection mediated variation in virulence can have positive effects on the host, ranging from the inhibition of the growth of certain parasites to the reduction of host mortality; the specific outcome depends on environmental conditions and the order in which the host is infected by the different parasites [78, 80, 174]. For example, when hepatitis B virus (HBV) and hepatitis C virus (HCV) co-infect a host, they inhibit each other’s replication. The existence of multiparasitism should also lead to the development of multi-therapy strategies that involve simultaneously treating all parasites present. For example, multi-targets drug can be more efficient against multiparasite strains [175]. It is also possible to take advantage of antagonistic interactions among certain parasites to fight parasites of medical importance [176]. This is the approach used in phage therapy, where bacteriophagous viruses are used to treat certain infectious diseases caused by bacteria [121, 177, 178]. It may also be possible to develop therapies that use virophages to treat infectious diseases of viral origin, given the discovery of subviral agents such as Sputnik, which is capable of inhibiting the growth of the mimivirus of the *Acanthamoeba polyphaga* protozoan [179, 180].

### Effects of co-infections on the transmission and diagnosis of infectious diseases

The different mechanisms that underlie interactions among parasites may result in a strong correlation between the incidences of two different diseases. This phenomenon is largely due to the fact that the presence of one or more parasites can affect, either positively or negatively, the infection probabilities of additional parasites, parasite transmission rates, and host susceptibility [181, 182]. Positive interactions among parasites are the best described and can contribute to disease emergence or re-emergence [183, 184]. A classic example is the increase in the incidence of two rare diseases—*Pneumocystis carinii*-induced pneumonia [185] and Kaposi sarcoma, a type of skin cancer [186, 187]—caused by HIV infection. HIV suppresses the host immune system, which allows other infections to take hold. This system is an example of a syndemic, *i.e.* the aggregation (synchronised epidemics) of two or more diseases that act synergistically [188]. To date, it has been discovered that Kaposi's Sarcoma is associated with another virus, in addition to the HIV, a Herpes virus [189]. Other syndemics have been described, such as associations among tick-vectored diseases such as borreliosis, babesiosis, and ehrlichiosis [190–192]. This particular syndemic results in variable, but often severe, clinical symptoms; consequently, it is frequently misdiagnosed and treated with unsuitable antimicrobial medicines. The syndemic produced by the pairing of influenza and tuberculosis causes high levels of mortality in affected populations. Adopting a perspective that takes multiparasitism into account may help, in certain cases, to identify the causes of disease outbreaks or declines and can thus inform the development of monitoring tools and surveillance methods [193]. In some cases, domestic animals could serve as sentinels for humans [194].

### The implications of multiparasitism for preventative measures used against infectious diseases

Because multiparasitism can have a major influence on parasite circulation, it is crucial to account for its effects when instituting disease prevention measures [20]. An inappropriate treatment can worsen the situation, for example, it has been highlighted that anthelmintic therapy can enhance the spread of co-infected microbial pathogens in some cases [195]. Even if their efficacy is altered by interactions among parasites [196–198], vaccination programs are nonetheless useful preventative measures for limiting the number of parasites to which a host is exposed [199]. They play an even more important role in limiting encounters among parasites that interact synergistically [200].

In addition to classical methods such as the use of insecticides or acaricides [201] or novel methods such as vaccines [199], it is possible to prevent vector-borne diseases by exploiting the antagonistic interactions among certain parasites to control the dispersal of vector-borne pathogens. For example, it has been found that, in *Aedes aegypti* mosquitoes, symbiotic *Wolbachia* bacteria limit the replication of dengue viruses, Chikungunya viruses, and malaria parasites [71–73]. Consequently, it may be possible to control the propagation of these three medically important pathogens by introducing *Wolbachia* into certain mosquito populations.

The inhibitory effects of multiparasitism can also be exploited to benefit hosts. For example, competition among parasites may limit the number of parasites that can infect a given host. In the food-processing industry, probiotics can be added to certain foods so as to limit the number of infectious intestinal pathogens and stimulate the host immune system [202]. These inhibitory effects are of paramount importance because they help prevent foods from being contaminated by parasites that pose major threats to human health [203, 204]. For example, the biofilms produced by *Staphylococcus sciuri* limit the growth of *Listeria monocytogenes*—the pathogen that causes listeriosis—as well as its adherence, especially to stainless steel surfaces. This interaction makes it possible to better control *L. monocytogenes* contamination in food-processing facilities [205].

## Conclusion

To more thoroughly understand the phenomenon of multiparasitism, it is necessary to develop analytical approaches that move past the one host-one parasite paradigm to adopt a multihost-multiparasite perspective [23, 24].

From this review, we can identify four major research directions that are aimed at clarifying the interactions taking place during co-infections. First, detecting community-level interactions is a methodological challenge that remains to be tackled [61]. Second, in order to better understand interactions among parasites, it will be necessary to make progress in identifying and accounting for common risk factors, as at present, it is complicated to incorporate risk factors into theoretical models [105, 107, 128]. Potential research paths already exist, namely, those that exploit network theory and association screening. However, it will be crucial to develop statistical tests that can used with network analyses. Third, progress needs to be made in incorporating non-independence among hosts (*e.g.* contagion) into analytical models, because multiparasitism affects parasite transmission dynamics. Fourth, it is important to put current biological findings to use in improving laboratory studies of multiparasitism [206]. The goal is to better mimic natural systems in order to identify and understand the real mechanisms underlying parasite interactions. In conclusion, in all future research, it will be essential to promote multidisciplinary approaches and collaborations with a view to improving our understanding of multiparasitism and its consequences.
